# Retracted: Treatment of Acute Pulmonary Embolism: Update on Newer Pharmacologic and Interventional Strategies

**DOI:** 10.1155/2019/4217864

**Published:** 2019-08-22

**Authors:** BioMed Research International

*BioMed Research International* has retracted the article titled “Treatment of Acute Pulmonary Embolism: Update on Newer Pharmacologic and Interventional Strategies” [1]. The article was found to contain a substantial amount of material, without citation, from previously published articles, including the following sources:

- William T. Kuo. “Endovascular Therapy for Acute Pulmonary Embolism”, Journal of Vascular and Interventional Radiology, 2012. https://doi.org/10.1016/j.jvir.2011.10.012. [2]
- Peter H. Lin, Huiting Chen, Carlos F. Bechara, and Panagiotis Kougias. “Endovascular Interventions for Acute Pulmonary Embolism”, Perspectives in Vascular Surgery and Endovascular Therapy, 2010. (http://journals.sagepub.com/doi/10.1177/1531003510379880). [3]
- Mitchell J. Daley, and Ishaq Lat. “Clinical Controversies in Thrombolytic Therapy for the Management of Acute Pulmonary Embolism”, Pharmacotherapy The Journal of Human Pharmacology and Drug Therapy, 2012. https://doi.org/10.1002/PHAR.1051. [4]
- Michael R. Jaff, M. Sean McMurtry, Stephen L. Archer, Mary Cushman, Neil Goldenberg, Samuel Z. Goldhaber, J. Stephen Jenkins, Jeffrey A. Kline, Andrew D. Michaels, Patricia Thistlethwaite, Suresh Vedantham, R. James White, Brenda K. Zierler, and on behalf of the American Heart Association Council on Cardiopulmonary, Critical Care, Perioperative and Resuscitation, Council on Peripheral Vascular Disease, and Council on Arteriosclerosis, Thrombosis and Vascular Biology, “Management of massive and submassive pulmonary embolism, iliofemoral deep vein thrombosis, and chronic thromboembolic pulmonary hypertension: a scientific statement from the American Heart Association”, Circulation, vol. 123, no. 16. (https://www.ahajournals.org/doi/full/10.1161/CIR.0b013e318214914f) https://doi.org/10.1161/CIR.0b013e318214914f. [5]
- David F. Allie, Chris J. Hebert, Raghotham R. Patlotla, Agostino Ingraldi, and Craig M. Walker: “Contrast-Induced Nephropathy & Targeted Renal Therapy During Peripheral Vascular Interventions: Results of the Louisiana Targeted Renal Therapy in Critical Limb Ischemia (TRT-CLI) Registry”, American Journal of Cardiology, Volume 104, Issue 6, p. 198D, article TCT-535. https://doi.org/10.1016/j.amjcard.2009.08.571. [6]
